# Role of YAP in hematopoietic differentiation and erythroid lineage specification of human-induced pluripotent stem cells

**DOI:** 10.1186/s13287-023-03508-z

**Published:** 2023-09-29

**Authors:** Chuti Laowtammathron, Chanchao Lorthongpanich, Nittaya Jiamvoraphong, Pimonwan Srisook, Phatchanat Klaihmon, Pakpoom Kheolamai, Sudjit Luanpitpong, Surapol Issaragrisil

**Affiliations:** 1 Siriraj Center of Excellence for Stem Cell Research, Department of Medicine, Faculty of Medicine Siriraj Hospital, Mahidol University, Bangkok, 10700 Thailand; 2 Center of Excellence in Stem Cell Research and Innovation, Faculty of Medicine, Thammasat University, Pathumthani, 12120 Thailand; 3 Division of Hematology, Department of Medicine, Faculty of Medicine Siriraj Hospital, Mahidol University, Bangkok, Thailand; 4 Bangkok Hematology Center, Wattanosoth Hospital, BDMS Center of Excellence for Cancer, Bangkok, Thailand

**Keywords:** YAP, iPSCs, Self-renewal, Differentiation, Hematopoietic stem cells, Erythropoiesis

## Abstract

**Background:**

In vitro production of hematopoietic stem/progenitor cells (HSPCs) from human-induced pluripotent stem cells (hiPSCs) provides opportunities for fundamental research, disease modeling, and large-scale production of HLA-matched HSPCs for therapeutic applications. However, a comprehensive understanding of the signaling mechanisms that regulate human hematopoiesis is needed to develop a more effective procedure for deriving HSPCs from hiPSCs.

**Methods:**

In this study, we investigate the role of YAP during the hematopoietic differentiation of hiPSCs to HSPCs and erythrocytes using the isogenic YAP-overexpressing (YAP-S5A) and YAP-depleting (YAP-KD) hiPSCs to eliminate the effects of a genetic background variation.

**Results:**

Although YAP is dispensable for maintaining the self-renewal and pluripotency of these hiPSCs, it affects the early cell-fate determination and hematopoietic differentiation of hiPSCs. Depleting YAP enhances the derivation efficiency of HSPCs from hiPSCs by inducing the mesodermal lineage commitment, promoting hematopoietic differentiation, and preventing the differentiation toward endothelial lineage. On the contrary, the overexpression of YAP reduced HSPCs yield by inducing the endodermal lineage commitment, suppressing hematopoietic differentiation, and promoting the differentiation toward endothelial lineage.

**Conclusions:**

Expression of YAP is crucial for the differentiation of hiPSC-derived HSPCs toward mature erythrocytes. We believe that by manipulating YAP activity using small molecules, the efficiency of the large-scale i*n vitro* production system for generating hematopoietic stem/progenitor cells for future therapeutic use could be improved.

**Supplementary Information:**

The online version contains supplementary material available at 10.1186/s13287-023-03508-z.

## Key point

The present study demonstrated that the dynamic expression of YAP is crucial for controlling the hematopoietic differentiation of hiPSCs. Suppression of YAP activity in hiPSCs enhances their hematopoietic differentiation resulting in more than a 100-fold increase in HSPC yield. The knowledge gained from this study can be used to improve the efficiency of HSPC derivation from hiPSCs for future therapeutic applications.

**Supplementary Information:**

The online version contains supplementary material available at 10.1186/s13287-023-03508-z.

## Introduction

Although the allogeneic hematopoietic stem cell transplantation (HSCT) has successfully been used to cure many debilitating hematological disorders, finding a proper matched hematopoietic stem/progenitor Cell (HSPCs) for each patient remains a significant hurdle. In vitro production of HLA-matched HSPCs from limitless cell sources such as induced pluripotent stem cells (iPSCs) could be a potential solution for such a problem. However, enhancing the efficiency of HSPC derivation from hiPSCs requires a better understanding of the signaling pathways that control the hematopoietic differentiation of iPSCs.

The Hippo signaling pathway was initially discovered in *Drosophila* through genetic screening and appeared highly conserved between flies and mammals [1]. Later studies showed that the Hippo signaling pathway plays a critical role in mammalian development during the pre- and post-implantation stages [2–4]. However, its functions in the self-renewal and differentiation of pluripotent stem cells, such as embryonic and induced pluripotent stem cells, remain controversial [5–12]. Focusing on hematopoiesis, Yorkie, a crucial transcription co-activator in the Hippo signaling pathway, has been shown to regulate the growth and differentiation of crystal cells, a *Drosophila* immune cell [13, 14]*.* Lundin and colleagues also reported the role of YAP (Yorkie homolog) in regulating zebrafish hematopoiesis [15].

In the mammalian system, YAP plays a role during mouse erythropoiesis as the conditional knockout of both YAP and TAZ, a YAP homolog, leads to the development of anemia in those mice [16, 17], while the overexpression of YAP causes the rapid expansion of mouse erythroid progenitors both in vivo and in vitro [18]. Our group and others have recently demonstrated an essential role of the YAP signaling pathway in human erytro- and megakaryopoiesis by showing that the expression level of YAP affects the proliferation and maturation of human erythrocytes and megakaryocytes [19–23]. Although the evidence regarding the roles of YAP in hematopoiesis has been accumulated, the roles of Hippo-YAP signaling pathway during other stages of human hematopoiesis have yet to be clearly elucidated.

In this study, we investigate the role of YAP during the hematopoietic differentiation of hiPSCs. Our study offers an insight into the impact of YAP on the derivation of HSPCs from hiPSCs and the hemato-endothelial lineage specification of those hiPSC-derived HSPCs. We believe that the insight gained from this study could be used to establish a more efficient i*n vitro* production system to generate iPSC-derived HSPCs for future therapeutic use.

## Materials and methods

### Culture of human-induced pluripotent stem cells (hiPSCs)

Human iPSC lines were cultured in Matrigel-coated plates in the Nutristem medium (Corning, USA). Cells were passaged every 5 days by treating with Versene (Thermo Fisher Scientific, USA) for 3–5 min, transferred to freshly prepared Matrigel-coated plates, and cultured under hypoxic conditions with 5% CO_2_ and 5% O_2_ at 37 °C.

### Establishment of the YAP-knockdown (YAP-KD) and YAP-overexpressing (YAP-S5A) isogenic hiPSCs

**Fig. 1:**
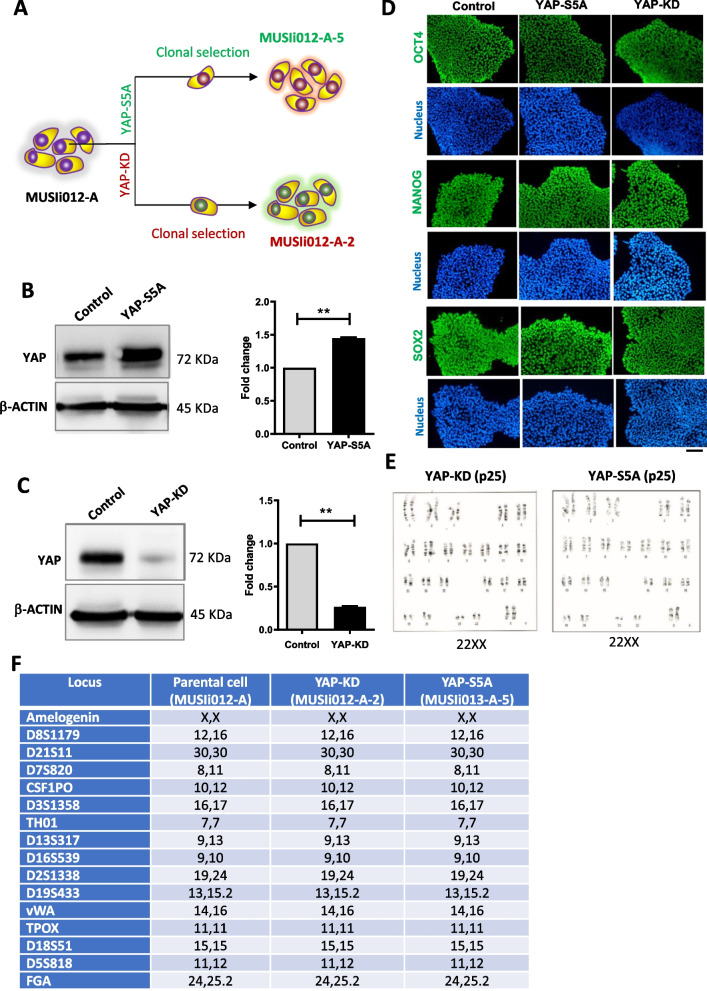
Expression of YAP does not alter the pluripotency of human iPSCs. **A** The schematic diagram demonstrated isogenic cell line establishment starting from a parental cell line (MUSIi012-A), YAP-depletion (YAP-KD; MUSIi012-A-2) and YAP-overexpressing (YAP-S5A; MUSIi012-A-4) cell lines. **B** Western blot results showed increasing of YAP in the YAP-S5A cell line, and **C** reduction of YAP in the YAP-KD cell line. Fold-change has been calculated from western blot band intensity and the presented in histograms. **D** Immunofluorescent staining for pluripotent markers OCT4, NANOG and SOX2 expression. **E** Normal karyotype (22xx) of the YAP-KD and YAP-S5A. F) STR analysis result comparing the parental, YAP-KD and YAP-S5A cell lines. Bar, 200 µm. Full-length blots are presented in Additional file 1: Fig. S1

The parental hiPSC line MUSIi012-A was transfected with a Crispr/Cas9 plasmid construct (PX459; Addgene, Cambridge, MA, USA) containing guide RNA targeting YAP1 using the lentiviral transfection system to generate YAP-KD hiPSCs. The guide RNA was designed by using a web-based sgRNA design tool (www.crispr.mit.edu) with minimal risk of off-target [5]. To generate the YAP-S5A hiPSCs, the MUSIi012-A cells were transfected with plasmids encoding the constitutively active YAP (YAP-S5A, a kind gift from Dr. Siew Wee Chan, Institute of Molecular and Cell Biology (IMCB), Singapore. At 24 h after transfection, the transfected cells were treated with 2 μg puromycin for 2 days to eliminate the non-transfected cells, and the remaining cells were subjected to single-cell cloning [5] (Fig. 1A). These procedures were applied to all iPSCs used in this experiment.

### Single-cell cloning of the YAP-KD and YAP-S5A isogenic hiPSCs

To generate single-cell clones, the transfected hiPSCs were washed twice with PBS before being treated with Accutase (Innovative Cell Technologies, AT104) to generate a single-cell suspension and resuspended in a Nutristem medium at the density of 20 cells/mL. One hundred microliters of singled-cells suspension was loaded into each well of a Matrigel-coated 96-well plate. The presence of a single cell in each well was confirmed under an inverted microscope, and mouse embryonic fibroblasts (MEFs) were seeded into each well on top of the singled cell. The colony that arose in each well was mechanically passaged using a glass-pulled pipette and further expanded in a Matrigel-coated plate under hypoxic conditions with 5% CO_2_ and 5% O_2_ at 37 °C. After screening, the successful manipulation cell lines were named as MUSI012-A-2 and MUSI012-A-5 for YAP-KD and YAP-S5A, respectively.

### Differentiation of hiPSCs to hematopoietic stem/progenitor cells (HSPCs)

The hiPSCs were treated with Accutase to generate a single-cell suspension and plated into an AggreWell^TM^800 24-well plate at a density of 2.16 × 10^5^ hiPSCs/well containing differentiation medium 1 (DM 1) is Stempro-34 medium supplemented with 2 mM L-glutamine, 400 μM monothioglycerol, 150 μg/ml transferrin and 50 μg/ml ascorbic acid] supplemented with 10 μM Y27632 and 10 ng/ml bone morphogenic protein-4 (BMP4). The following day, the EBs that arise in each well were transferred to a six-well plate and cultured in DM 1 supplemented with 10 ng/ml BMP4, 5 ng/ml basic fibroblast growth factor (bFGF), and 3 μM CHIR99021. On culture day 3, the EBs were transferred into Matrigel-coated twelve-well plates in differentiation medium II [DM 2 is DM 1 supplemented with 5 ng/ml bFGF, 15 ng/ml vascular endothelium growth factor (VEGF), 30 ng/ml IL-3, 10 ng/ml IL-6, 5 ng/ml IL-11, 25 ng/ml insulin-like growth factor 1 (IGFI), 50 ng/ml stem cell factor (SCF), 2 U/ml erythropoietin (EPO), 30 ng/ml thrombopoietin (TPO), 10 ng/ml FMS-like tyrosine kinase ligand (FLT3L)]. On culture day 6, the media were replaced with differentiation medium III [DM 3 is DM 2 supplemented with 10 ng/ml BMP4]. From culture day 0 to day 8, the cells were cultured under a hypoxic atmosphere containing 5% O_2_ and 5% CO_2_. On culture day 9, cells were cultured in a normoxic atmosphere containing 5% CO_2_ until the end of culture (culture day 13). The expression of HSPC markers, CD34, CD43, and CD45, were determined by flow cytometry on culture days 6, 9, and 13. At the end of the culture, the derived HSPCs were sorted and subjected to erythroid differentiation.

### Differentiation of hiPSCs-derived HSPCs to erythroid cells

The hiPSCs-derived HSPCs were cultured using a three-stage erythroid differentiation procedure which has been established and described in Griffiths et al. [24]. The basal medium was Iscove’s modified Dulbecco’s medium (IMDM, #FG0465; Biochrom Ltd, UK) supplemented with 2% (v/v) heat-inactivated fetal bovine serum (FBS; Merck Millipore, USA), 3% (v/v) heat-inactivated human AB serum, 200 μg/ml transferrin (T0665; Sigma-Aldrich, USA), 3 U/ml heparin (Leo Pharma, Denmark), 10 μg/ml insulin (I9278; Sigma-Aldrich, USA), 3 U/ml EPO (Janssen Pharmaceutica, Belgium), 100 U/ml of penicillin (Sigma-Aldrich, USA), and 100 mg/ml streptomycin (Sigma-Aldrich, USA). For stage I (day 0–8), the basal medium was supplemented with 10 ng/ml Stem Cell Factor (SCF; R&D Systems, USA) and 1 ng/ml interleukin-3 (IL-3; R&D Systems, USA). For stage II (day 8–11), the basal medium was supplemented with 10 ng/ml SCF, and for stage III (day 11 onward), the basal medium was supplemented with 500 μg/ml transferrin. On culture day 0, the HSPCs derived from hiPSCs were seeded at a density of 2 × 10^5^ cells/ml and maintained at a density of 2–5 × 10^5^ cells/ml in a humidified atmosphere with a 5%CO_2_ at 37 °C. The medium was replaced every 3 days. On culture day 8, the cells were harvested, resuspended in stage II medium, and cultured at a density of 5 × 10^5^ cells/ml. On culture day 11, the cells were harvested, resuspended in stage III medium, and cultured at a density of 5–10 × 10^5^ cells/ml. At this stage, the medium was replaced every day until the end of culture (culture day 13).

### Transcription analysis

The isolated total RNA was reverse-transcribed using the High-Capacity cDNA Reverse Transcription Kit (Applied Biosystems, Foster City, CA, USA). Quantitative real-time polymerase chain reaction (PCR) was performed using Realtime PCR Master Mix (Applied Biosystems, USA) and the Universal Probe Library (UPL; Roche Life Science, Germany) in a final volume of 10 µl. Real-time PCR was performed using a CFX384 Touch Real-Time PCR Detection System (Bio-Rad Laboratories, Hercules, CA, USA). A list of the primers used in this study is provided in Additional file 2: Table S1.

### Cytospin and wright’s staining

Cells were harvested and spun onto a glass slide at 1000 rpm for 5 min using a Cytospin centrifuge (Thermo Fisher Scientific, USA). Wright’s staining solution was dropped onto the glass slide, followed by an equal volume of distilled water, and the solution was then mixed by gentle blowing. Cells were incubated for 4 min, washed extensively with tap water, and left to dry. The mounting medium (Fisher Chemical, USA) was applied, and a glass coverslip was placed over the stained cells. The cell morphology was observed under a light microscope (Olympus Microscope CX31; Olympus, Tokyo, Japan).

### Short tandem repeat (STR) analysis

The YAP-KD and YAP-S5A hiPSCs were subjected to STR analysis to confirm their isogenic identity to their parental MUSIi012-A cells at the Department of Forensic Medicine, Faculty of Medicine Siriraj Hospital, Mahidol University, Thailand.

### Karyotyping

The YAP-KD and YAP-S5A hiPSCs were subjected to standard G-banding karyotyping analysis at the Division of Medical Genetics, Department of Obstetrics & Gynecology, Faculty of Medicine Siriraj Hospital, Thailand.

### Immunofluorescence staining

Immunofluorescence staining was performed as previously described [25]. Briefly, cells were fixed with 4% (w/v) paraformaldehyde for 30 min at room temperature, washed with PBS, incubated with 0.1% (w/v) Triton-X 100 in PBS for 30 min, and blocked with 10% (v/v) FBS for 1 h at room temperature. At this stage, the primary antibodies against human OCT4 (Cell Signaling Technology; 1:300 dilution), human NANOG (Thermo Fisher Scientific; 1:100 dilution), and human SOX2 (Millipore; 1:100 dilution) were added, and the cells were incubated overnight. Cells were then washed twice with PBS and incubated with goat anti-rabbit antibody conjugated with Alexa Fluor 488 (Thermo Fisher Scientific; 1:500 dilution) for 1 h in the dark. Nuclei were counterstained with Hoechst 33,342 (Thermo Scientific, MA, USA; 1:1000 dilution). The fluorescence images were acquired using a Nikon Eclipse Ti-U Inverted Fluorescence Microscope (Nikon Corporation, Tokyo, Japan).

### Western blot analysis

Total protein was isolated from cells using a protein lysis buffer (10 × RIPA: Cell Signaling Technology, USA) supplemented with protease inhibitors (Roche Life Science, USA). The electrophoresis was performed using 7–12% SDS/polyacrylamide gels. The separated proteins were transferred to PVDF membranes (Merck Millipore, Germany) and incubated with the antibody against human YAP (Cell Signaling Technology; 1:1000 dilution) and human β-ACTIN (Sigma-Aldrich, 1:25,000 dilution) for 16 h. The membranes were then washed and incubated with the appropriate peroxidase-conjugated secondary antibodies for 1 h. The resulting autoradiography was then detected by enhanced chemiluminescence (Merck Millipore, Germany).

### Flow cytometry

The cells were harvested, blocked with 2% (w/v) bovine serum albumin in phosphate buffer saline (PBS) for 30 min, and incubated with the appropriate fluorescent-labeled antibodies at room temperature for 15 min in the dark. A list of the antibodies used is provided in Additional file 3: Table S2. All antibodies were used at a dilution of 1:50. After incubation with the antibodies, cells were washed with FACS buffer (PBS + 2% Bovine Serum Albumin (BSA); Sigma-Aldrich), fixed with 1% (w/v) paraformaldehyde, and kept at 4 °C until used. The flow cytometry was performed using BD FACSCanto Flow Cytometer (BD Biosciences, Franklin Lakes, NJ, USA).

### Colony-forming assay

5 × 10^4^ HSPCs were resuspended in 100 µl MethoCult™ medium (Stemcell technologies) and gently dispensed into a 35-mm dish using a 1-mL syringe. Cells were cultured at 37 °C with 5% CO_2_. Colony counting and classification were performed after 14 days of culture under an inverted microscope.

### Statistical analysis

The results are presented as mean ± standard deviation (SD). Mann–Whitney U test was used to compare nonparametric variations between groups. A *p*-value of < 0.05 was considered to be statistically significant. The data were analyzed by GraphPad Prism software version 8.0 (GraphPad Software, USA, www.graphpad.com).

## Results and discussion

### Establishment of YAP-overexpressing- and YAP-knockdown hiPSCs

After genetic manipulation, the expression level of *YAP* in the YAP-overexpressing- (YAP-S5A) and YAP-knockdown (YAP-KD) hiPSCs was determined. As expected, the level of YAP protein in the YAP-S5A hiPSCs was significantly enhanced 1.45-fold compared to control (Fig. 1B), while the YAP level in the YAP-KD hiPSCs was 4.0-fold depleted compared to control (Fig. 1C and full-length blots are presented in Additional file 1: Fig. S1). Both YAP-S5A and YAP-KD hiPSCs express all the pluripotency marker proteins, OCT4, NANOG, and SOX2, similar to that of their unmanipulated counterpart (Fig. 1D). Moreover, YAP-S5A and YAP-KD hiPSCs also exhibited stable diploid karyotypes (46, XX) even after being expanded for 25 passages (Fig. 1E). These results suggest that the manipulation of YAP expression did not alter the pluripotency or cause the genetic instability of the iPSCs. A short tandem repeat (STR) analysis confirms the isogenic identity of YAP-KD and YAP-S5A hiPSCs to their parental cell line, MUSIi012-A (Fig. 1F). In addition, teratoma formation was performed to determine in vivo differentiation capacity of both YAP-KD and YAP-S5A. Representative cells from the three—embryonic germ layers were found in teratoma derived from all cell lines (Additional file 1: Fig. S2).

### Depletion of YAP reduced the ability of hiPSCs to form embryoid body (EB)

**Fig. 2:**
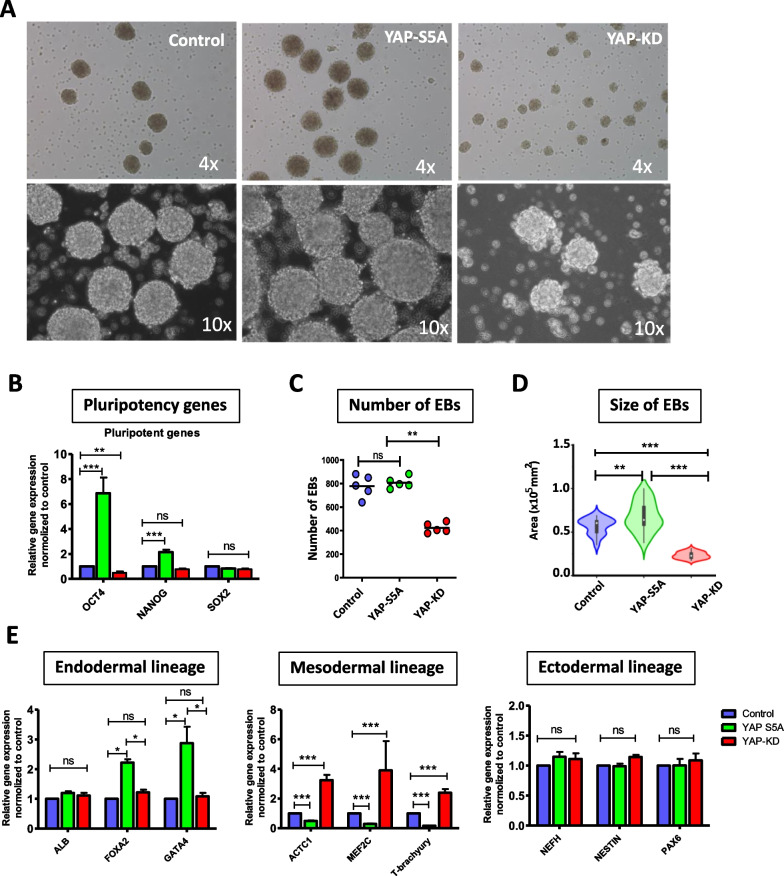
Effects of gene manipulations on YAP relative to in vitro differentiation. **A** Embryoid body formation of control, YAP-S5A and YAP-KD cell lines (Upper row: 4 × magnification, and Lower row: 10 × magnification). **B** Pluripotent genes expression, **C** Number of EBs (each dot represents each replication), **D** Size of EBs obtained from the single-cell aggregation of each cell line. **E** Transcription analysis of endoderm, mesoderm, and ectoderm specific markers. Quantitative data are presented as mean ± SEM; n = 5 otherwise stated, Mann–Whitney U test, *p*-value of ***p < 0.001, **p < 0.01, *p < *0.05*, ns = no significant difference

To determine whether the alteration of YAP affects an in vitro differentiation of hiPSCs, YAP-S5A and YAP-KD hiPSCs was subjected to EB formation assay (Fig. 2A). On day 3, EBs from all treatments were observed and collected for further analysis. The YAP-S5A expressed higher levels of pluripotent marker genes, *OCT4 and NANOG*, while the YAP-KD expressed lower levels of *OCT4* than the control MUSIi012-A cells (Fig. 2B). Although the overexpression of YAP did not significantly alter the EB forming capacity of the YAP-S5A hiPSCs compared with the control (808 ± 48 vs. 778 ± 95; *P* > *0.05*), the depletion of YAP significantly reduced the EB forming capacity of YAP-KD hiPSCs compared with the control (422 ± 42 vs. 778 ± 95, *p* < *0.01*) (Fig. 2C). Moreover, the EBs derived from YAP-KD hiPSCs were significantly smaller, while the EBs derived from YAP-S5A hiPSCs were relatively larger than the control (Fig. 2Aand D). To study the effect of YAP on the early lineage specification of hiPSCs, the expression levels of endodermal, mesodermal, and ectodermal genes in day 3 EBs derived from YAP-S5A, and YAP-KD hiPSCs were determined. The results showed that the EBs derived from YAP-S5A hiPSCs expressed significantly higher levels of endodermal genes but lower levels of mesodermal genes than the control (Fig. 2E). On the contrary, the EBs derived from YAP-KD hiPSCs expressed a significantly higher level of mesodermal genes than the control (Fig. 2E). These results suggest that YAP overexpression induces hiPSC differentiation toward endodermal cells, while the depletion of YAP leads to the differentiation of those cells toward mesodermal lineage.

### YAP activity influences the hematopoietic lineage commitment of human iPSCs

**Fig. 3:**
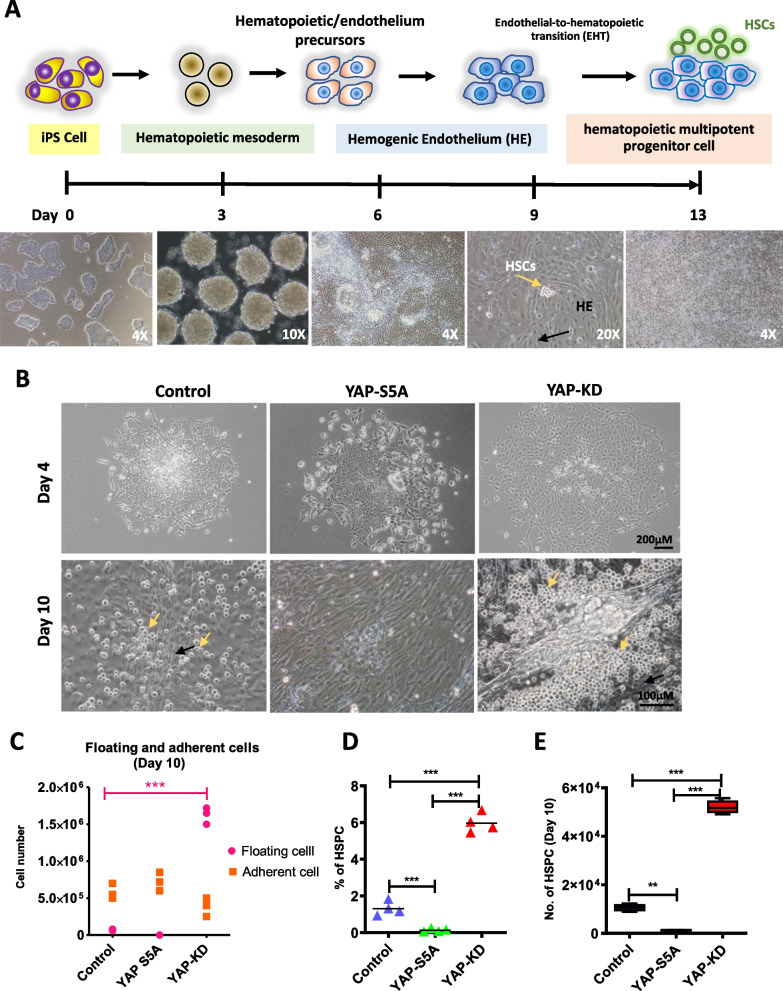
Effect of YAP on in vitro differentiation of human iPSCs to hematopoietic stem cells. **A** Schematic diagram of the protocol used to generate HSPCs from iPSCs in vitro. **B** Morphologies of differentiated EBs derived from control, YAP-S5A and YAP-KD cell lines on day 4, 8 and 10 after being plated on Matrigel. The yellow arrow indicates Hematopoietic Stem Cells (HSCs), black arrow indicates Hemogenic cells Endothelial (HE). **C** Histogram showing the number of, **D** total counts of floating and adherence cells Percentage of HSPCs, and **E** Number of HSPCs harvested from both floating and adherent cells at day 10 of culture. Each dot represents individual replication. All experiments were performed at least three times independently with the technical triplicate, and data were expressed as mean ± SEM, Mann–Whitney U test, the *p*-value of ***p < 0.001, **p < 0.01, *p < *0.05*

**Fig. 4:**
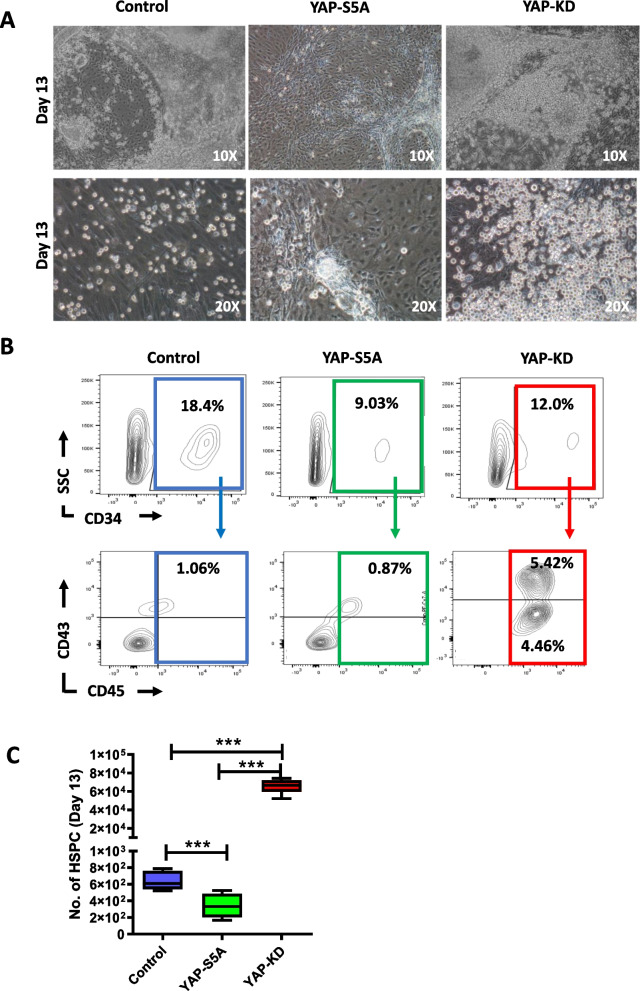
Effect of YAP on Hematopoietic Stem/Progenitor cells production yield. **A** Morphologies of differentiated EBs derived from control, YAP-S5A and YAP-KD cell line on day 13 after plated on Matrigel. The upper and lower row shows images taken at 10 × and 20 × magnification, respectively. **B** A representative histogram from Flow cytometry results showing cells expressing CD34, CD43 and CD45 on day 13 of differentiation. **C** Histogram shows the number of HSPCs collected from control, YAP-S5A and YAP-KD treatment on day 13. All experiments were performed at least three times independently with the technical triplicate, and data were expressed as mean ± SEM, ***p < *0.001*

To determine the effect of YAP on the hematopoietic differentiation of human iPSCs, YAP-S5A and YAP-KD hiPSCs were subjected to the hematopoietic differentiation procedure described in Fig. 3A. After 4 days of culture, the homogenous hemato-endothelial microarchitecture was observed among the differentiated YAP-KD hiPSCs, while the YAP-S5A hiPSCs and control cells failed to generate such structure (Fig. 3B). On culture day 10, several clusters of hematopoietic and hemogenic endothelial cells were observed in the differentiated YAP-KD hiPSCs, while none was observed in the differentiated YAP-S5A hiPSCs and control (Fig. 3B). Some differentiated hiPSCs detached from the culture surface and became floating hematopoietic cells. The differentiated YAP-KD hiPSCs generated more floating cells than the differentiated YAP-S5A hiPSCs and control (Fig. 3C). Consistent with this, the differentiated YAP-KD hiPSCs generated significantly higher percentage of HSPCs (CD34^+^/ CD43^+^/CD45^±^ cells) than control (5.96% ± 0.45 vs 1.3% ± 0.32; *p* < *0.001*) (Fig. 3D). On culture day 10, the number of HSPCs generated from YAP-KD hiPSCs were approximately fivefold higher than control (5.2 × 10^4^ ± 2,413 cells vs 1 × 10^4^ ± 1,296 cells; *p* < *0.001*) (Fig. 3E). On the contrary, the differentiated YAP-S5A hiPSCs generated lower percentage of HSPCs compared with control (0.38% ± 0.41 vs 1.3% ± 0.32; *p* < *0.001*) (Fig. 3D). The number of HSPCs derived from YAP-S5A hiPSCs on culture day 10 was also tenfold lower than control (0.1× 10^4^ ± 167 cells vs 1 × 10^4^ ± 1,296 cells; *p* < *0.01*) (Fig. 3E). Similar to the results observed on culture day 10, the YAP-KD hiPSCs generated a significantly higher percentage of HSPCs *(p* < *0.001),* while the YAP-S5A hiPSCs generated a lower percentage of HSPCs than control at the end of the culture *(p* < *0.01)* (Fig. 4A,B). At the end of the culture (culture day 13), the number of HSPCs derived from the YAP-KD hiPSCs (6.5 × 10^4^ ± 3.3 × 10^3^ cells) was approximately 100-fold higher than control (6.4 × 10^2^ ± 45 cells), while those generated from the YAP-S5A hiPSCs (1.46 × 10^2^ ± 60 cells) were twofold lower than control (Fig. 4C). These results suggest that the YAP level is crucial for the hematopoietic differentiation of hiPSCs, in which the excessive expression of YAP inhibits HSPC formation.

To determine whether these results are cell line dependent, an additional set of human iPSC line (MUSIi017A) with their YAP-manipulated subclones MUSIi017-A-1 (YAP-KD) and MUSIi017-A-2 (YAP-overexpressing) were subjected to HSPCs differentiation. The consistency results were found as the increasing of HSPCs formation was found in YAP-KD cell line while the inhibition was observed in the YAP-overexpressing cell line (Additional file 1: Fig. S3). These results suggest the role of YAP in a non-cell line dependent.

### YAP affects the hemato-endothelial lineage diversification of hiPSCs

**Fig. 5:**
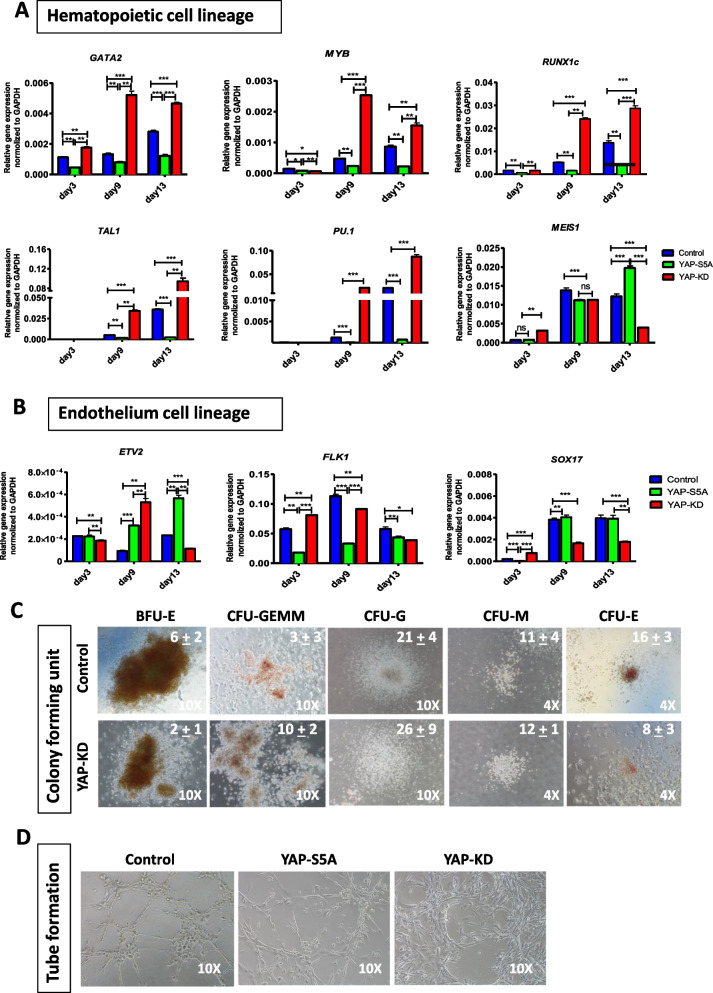
Effect of YAP on hemato-endothelium lineage segregation. **A** Histograms show transcripts of hematopoietic cell lineage markers and **B** Endothelium cell lineage markers. **C** The morphology of hematopoietic CFUs growing from HSPCs. Photos were illustrated at 4X and 10X magnification. **D** Representative pictures of the cells subjected to capillary-tube formation assay photographed at 12 h after plated. All experiments were performed at least three times independently with the technical triplicate, and data were expressed as mean ± SEM, ***p < *0.001*

Hemato-endothelial lineage diversification is one of the critical steps of HSPC differentiation. To investigate the role of YAP in such a process, we determined the expression levels of several hematopoietic and endothelial marker genes during the hematopoietic differentiation of the YAP-KD and YAP-S5A hiPSCs. Throughout the differentiation, the differentiated YAP-KD hiPSCs expressed much higher levels of five hematopoietic markers, *GATA2, MYB, RUNX1C, TAL1,* and *PU.1*, than the control cells, while the expression levels of these markers in the differentiated YAP-S5A hiPSCs were lower than control (Fig. 5A).

On the contrary, the expression levels of three endothelial markers, *ETV2, FLK,1,* and *SOX17*, in the differentiated YAP-KD iPSCs were significantly lower than those of control at the end of the culture. In contrast, the differentiated YAP-S5A iPSCs expressed a significantly higher level of *ETV2* than the control at the end of the culture (Fig. 5B). Similar to the HSPCs derived from control cells, the HSPCs derived from YAP-KD iPSCs formed all types of myeloid and erythroid colonies when subjected to methylcellulose colony-forming assay. Number of colonies arose were counted and indicated in the picture (Fig. 5C). The colony-forming assay on the HSPCs derived from YAP-S5A iPSCs could not be performed due to an insufficient number of the derived HSPCs. Moreover, when the endothelial cells (CD34^+^/144^+^cells) derived from the YAP-KD iPSCs were subjected to an in vitro tube formation assay, they failed to form a capillary-like structure (Fig. 5D), while those derived from the YAP-S5A iPSCs and controls formed several capillary-like structures (Fig. 5D). These results suggest that expression of YAP affects the hemato-endothelial lineage diversification of iPSCs by promoting hematopoietic differentiation while preventing endothelial lineage commitment.

It has been reported that Notch signaling is crucial for mammalian hematopoietic development and HSPC differentiation [26, 27]. Constitutively expression of Notch1-intracellular domain (N1-ICD) results in increasing self-renewal and differentiation capacity of HSPCs [28]. Recent studies suggest that the crosstalk between Hippo-YAP and Notch pathways is critical for maintaining the balance between progenitor maintenance and cell differentiation in different tissues but yet iPSCs-derived HSPCs [29–31]. To determine whether YAP influences in vitro production of iPSC-derived HSPCs via Notch signaling, HSPCs from control and YAP-KD hiPSCs were sorted and subjected for determining N1-ICD active Notch ligand expression concurrently with its non-HSPCs of the same differentiation cohort. Result showed N1-ICD is highly expressed in HSPCs but not non-HSPCs population. Interestingly, HSPCs-derived from YAP-KD hiPSCs showed significantly upregulated of N1-ICD compared to the control-derived HSPCs (Additional file 1: Fig. S4A and full-length blots are presented in Additional file 1: Fig. S4B). This result clearly suggests that YAP acts as a negative regulator for N1-ICD expression in HSPCs. Reduction of YAP enhances Notch signaling activity by upregulating N1-ICD resulting in increasing HSPCs formation.

### YAP is required for normal human erythropoiesis

**Fig. 6:**
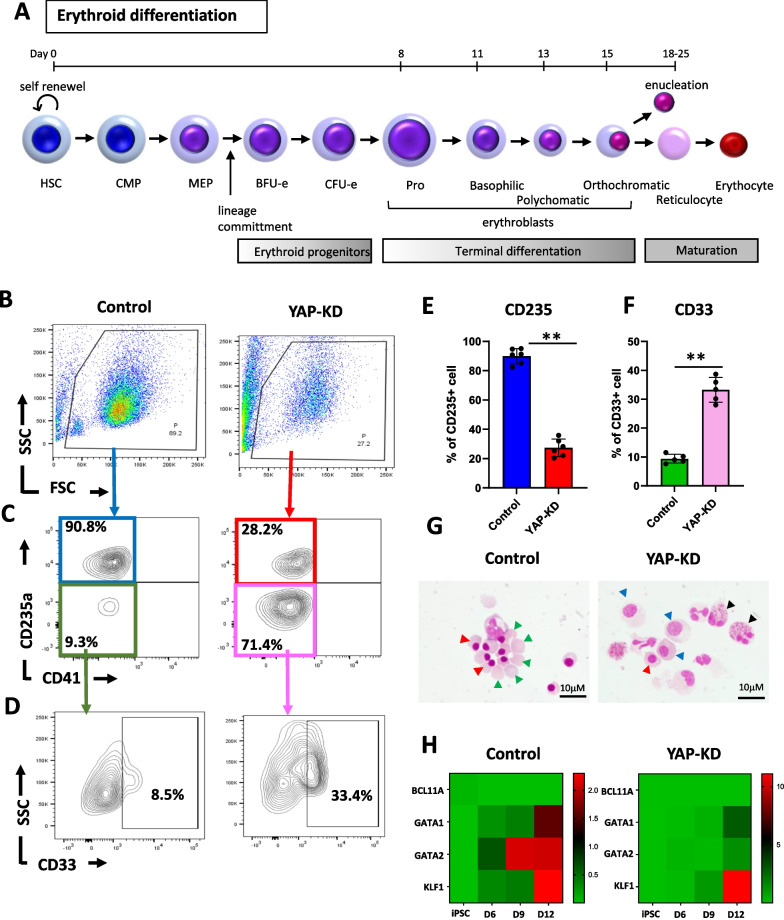
Reduction of YAP inhibits erythropoiesis. **A** Schematic diagram of the protocol used to generate erythroid cells from iPSC-derived HSPCs in vitro. **B**, **C** A representative histogram from Flow cytometry results showing cells harvested from control and YAP-KD HSPCs expressing CD235 erythroid marker but negative to CD41 megakaryocyte marker on day 18 of differentiation. **D** Flow cytometry analysis showing CD33 granulocyte marker expression. **E**, **F** Histograms show the percentage of CD235 and CD33 positive cells in control and YAP-KD HSPCs after being cultured in erythroid differentiation media for 18 days. **G** A representative cytospin pictures of the cells derived from control and YAP-KD HSPCs on day 13 of culture in erythroid differentiation media. Orthochromatic (red arrowhead), reticulocyte stage (green arrowhead), polychromatic (blue arrowhead), and granulocyte-like cells (black arrowhead) were indicated. **H** Heatmap showing the expression pattern of the late stage of erythroid-specific genes in control and YAP-KD HSPCs after cultured in erythroid differentiation media for 12 days. All experiments were performed at least three times independently with the technical triplicate, and data were expressed as mean ± SEM, **p < *0.01*

To further determine whether YAP is essential during the later stages of human hematopoiesis, the HSPCs derived from YAP-KD hiPSCs were subjected to the erythroid differentiation procedure described in Fig. 6A. At the end of culture, the YAP-KD hiPSCs generated fewer cells (Fig. 6B), produced much lesser percentage of erythroid cells (CD235^+^/CD41^−^) (28.2% vs 90.8%; *p* < *0.01*) (Fig. 6B and E) but generated higher percentage of myeloid cells (CD235^−^/CD41^−^/CD33^+^ cells) (33.4% vs 8.4%; *p* < *0.01*) than control cell (Fig. 6D and F). Moreover, most YAP-KD hiPSCs-derived erythroid cells were polychromatic erythroblasts which are less mature than the orthochromatic erythroblasts and reticulocytes derived from control hiPSCs cultured under the same condition (Fig. 6G). The subsequent transcriptional analysis also showed that the HSPCs derived from YAP-KD hiPSCs failed to up-regulate the expression levels of *GATA1, GATA2, and BCL11A,* which are critical for the erythroid maturation (Fig. 6H). They also expressed lower levels of *RUNX1, TAL1 LMO2, and PU.1* associated with the early stages of erythroid differentiation (Additional file 1: Fig. S5A,B). Analysis of globin genes expression showed that significant lower expression of both alpha and beta globin genes was found in YAP-KD iPSC-derived erythroid cells compared to control (Additional file 1: Fig. S5C; *p* < *0.001*). These results suggest that YAP plays an essential role in the differentiation of HSPCs toward mature erythrocytes. The depletion of YAP prevents erythroid differentiation.

hiPSCs are widely recognized as a promising cell source for regenerative medicine. While constructing a complex three-dimensional organ from stem cells is still out of reach, generating various hematopoietic cells from cultured stem cells for therapeutic use could probably be accomplished. Although several methodologies have been used to derive HSPCs from hiPSCs, the efficiency of those derivation methods is generally low, resulting in an insufficient number of HSPCs for therapeutic application. A better understanding of the signaling mechanisms regulating the hematopoietic differentiation of hiPSCs is required to improve the efficiency of HSPC derivation from hiPSCs. Although there is increasing evidence for the critical roles of the Hippo signaling pathway in animal hematopoiesis, the roles of YAP during the various stages of human hematopoiesis are yet unknown.

To investigate this, we first established the isogenic YAP-overexpressing and YAP-depleted hiPSCs (YAP-S5A and YAP-KD, respectively) from the same parental hiPSCs (MUSIi012-A cells) to eliminate genetic background variation and subjected them to hematopoietic differentiation through EB formation. Although the manipulation of YAP does not affect the ability of hiPSCs to form EBs, it affects the early cell-fate determination of hiPSCs. While YAP overexpression induces hiPSCs differentiation toward endodermal cells, YAP depletion causes hiPSCs to differentiate into mesodermal cells. This is in agreement with our previous study, which demonstrated that YAP and TAZ are dispensable for maintaining the pluripotency of hiPSCs [5, 6], but essential for their differentiation toward megakaryocytes, a mesodermal derivative [19, 20].

The YAP levels also affect the subsequent stages of hematopoietic differentiation, in which the excessive expression of YAP inhibits HSPC formation while the YAP depletion enhances hematopoietic differentiation of hiPSCs and improves the yield of HSPCs to more than 100-fold in comparison with those derived from normal hiPSCs under the same condition (Fig. 4C). Our results suggest that YAP depletion increases the efficiency of HSPC derivation by promoting hematopoietic differentiation while suppressing endothelial lineage commitment.

A recent cell–cell contact analysis reported by Xin and colleagues showed that the highest cell–cell contact signal in EBs was found in iPSC-derived endothelial cells, while the lower signal was found in the iPSC-derived hematopoietic cells [32]. It is worthy to note that YAP-S5A hiPSCs, that overexpressed YAP generated larger-sized EBs, while the EBs derived from YAP-KD hiPSCs, which expressed a lower level of YAP, were smaller than those derived from normal hiPSCs (Fig. 2A and D). The lower level of cell–cell contacts in the small EBs derived from YAP-KD hiPSCs might partially contribute to their differentiation toward hematopoietic lineages, while the higher level of cell–cell contacts in the larger EBs derived from YAP-S5A hiPSCs might drive their differentiation toward endothelial lineage.

Although YAP depletion could greatly enhance the efficiency of HSPC derivation from hiPSCs, those HSPCs could not further differentiate into mature erythrocytes (Fig. 6Gand H). These results suggest that YAP is essential during the later stages of HSPC differentiation, especially to the erythroid lineage. This result agrees with the previous study showing that the conditional knockout of both YAP and TAZ leads to the development of anemia in mice [16, 17]. It is worth noting that, when subjected to the megakaryocyte-inducing condition, the YAP-KD hiPSCs generated a higher number of megakaryocytes (CD41^+^ cells) than normal hiPSCs. This result suggests that the depletion of YAP might suppress the erythroid differentiation of hiPSCs by promoting megakaryocyte lineage commitment and production (Additional file 1: Fig. S6). However, further study is required to explore the insight mechanism underlying the role of YAP in hiPSCs-derived megakaryocytes.

New blood vessel formation is an essential physiological process seen in development. Our result also demonstrated that YAP is essential for human endothelial tube formation as the iPSC depleted YAP failed to form a capillary-like structure. This result is corresponding to previous reports demonstrating that YAP is an important regulatory protein regulating angiogenesis and vascularization in zebrafish and mouse [33–36].

Collectively, we found that the dynamic expression of YAP is crucial for the hematopoietic differentiation of hiPSCs. A reduction of YAP is needed to promote the differentiation of hiPSCs toward HSPCs, while the re-expression of YAP is required for the further differentiation of HSPCs toward mature erythrocytes.

## Conclusion

According to this study, which used hiPSCs and their YAP-manipulated isogenic cells as a model, the expression of YAP is one of the crucial elements that regulate hematopoietic lineage acquisition and differentiation of hiPSCs. We found that lowering the YAP level enhances the derivation efficiency of HSPCs from hiPSCs by inducing the mesodermal lineage commitment, promoting hematopoietic differentiation, and preventing the differentiation toward endothelial lineage. On the contrary, the overexpression of YAP reduced HSPCs yield by inducing the endodermal lineage commitment, suppressing hematopoietic differentiation, and promoting the differentiation toward endothelial lineage. However, YAP activity is crucial for the further differentiation of hiPSC-derived HSPCs toward erythroid lineage. We believe that by manipulating YAP activity using small molecules, the efficiency of the large-scale i*n vitro* production system for generating HSPCs for future therapeutic use could be improved.

### Supplementary Information

**Additional file 1**: **Fig. S1**. A representative photograph of an entire western blot membrane probed with YAP and ACTIN specific antibodies. The areas indicate the results used in Figures 1B and 1C, respectively. **Fig. S2**. H&E stained images of teratoma derived from human iPSC lines used in this study. A and B) Staining of teratoma derived from MUSIi012-A and MUSIi017-A and their YAP-manipulated subclones to determine representative cells of the 3-embryonic germ layers, including ectoderm, mesoderm and endoderm. **Fig. S3**. Number of hematopoietic progenitor cell (HSPCs) produced from MUSIi017-A (control), MUSIi017-A-1 (YAP-KD), MUSIi017-A-2 (YAP-overexpressing) as determined at day 10 of differentiation. **Fig. S4**. A) Expression of N1-ICD in non-Hematopoietic Progenitor cell (Non-HSPCs) and HSPCs derived from MUSIi012-A control and MUSIi012-A-2 (YAP-KD) cell lines. B) Representative photographs of the entire western blot membrane probed with N1-ICD and ACTIN specific antibodies. (C) Multiple exposures of the same blot shown in A. The areas indicate the results used in supplementary figure 4A. **Fig. S5**. A and B) Heatmap showing the expression pattern of erythroid induction genes in control and YAP-KD HSPCs after cultured in erythroid differentiation media for 18 days. C) Relative expression of globin to GAPDH in erythroid derived-IPSCs, Data were collected from at least three times independently with the technical triplicate, and data were expressed as mean ± SEM, ***P < 0.001. Supplementary figure 6: Number of CD41+ cells megakaryocyte and platelets production from MUSIi012-A-2 (YAP-KD) iPSCs compared to MUSIi-012-A (control). The measurement was done at 6 timepoints during the differentiation in sequential differentiation medium.

**Additional file 2**: **Table S1**. List of primers used in this study.

**Additional file 3**: **Table S2**. List of antibodies used in this study.

## Data Availability

No additional information was deposited elsewhere. All the data necessary for result interpretation are shown in this manuscript and supplementary files.

## Abbreviations

YAP: Yes-associated protein
KD: Knockdown
iPSC: Induced pluripotent stem cell
EBs: Embryoid bodies
HSPCs: Hematopoietic stem/progenitor cells
qRT-PCR: Quantitative reverse transcriptase-polymerase chain reaction

## References

[1] Pan D (2010). The hippo signaling pathway in development and cancer. Dev Cell.

[2] Lorthongpanich C (2013). Temporal reduction of LATS kinases in the early preimplantation embryo prevents ICM lineage differentiation. Genes Dev.

[3] Nishioka N (2009). The Hippo signaling pathway components Lats and Yap pattern Tead4 activity to distinguish mouse trophectoderm from inner cell mass. Dev Cell.

[4] Sharma J, Madan P (2020). Characterisation of the Hippo signalling pathway during bovine preimplantation embryo development. Reprod Fertil Dev.

[5] Lorthongpanich C (2020). YAP-depleted iPSC MUSIi012-A-2 maintained all normal stem cell characteristics. Stem Cell Res.

[6] Lorthongpanich C (2020). Generation of a serine/threonine-protein kinase LATS1 gene-edited iPSC MUSIi012-A-3. Stem Cell Res.

[7] Li P (2013). Functional role of Mst1/Mst2 in embryonic stem cell differentiation. PLoS ONE.

[8] Aylon Y (2014). Lats2 is critical for the pluripotency and proper differentiation of stem cells. Cell Death Differ.

[9] Chung H (2016). Yap1 is dispensable for self-renewal but required for proper differentiation of mouse embryonic stem (ES) cells. EMBO Rep.

[10] Lorthongpanich C (2019). Generation of a WWTR1 mutation induced pluripotent stem cell line, MUSIi012-A-1, using CRISPR/Cas9. Stem Cell Res.

[11] Lian I (2010). The role of YAP transcription coactivator in regulating stem cell self-renewal and differentiation. Genes Dev.

[12] Qin H (2016). YAP induces human naive pluripotency. Cell Rep.

[13] Milton CC (2014). The Hippo pathway regulates hematopoiesis in *Drosophila melanogaster*. Curr Biol.

[14] Ferguson GB, Martinez-Agosto JA (2014). Yorkie and Scalloped signaling regulates Notch-dependent lineage specification during *Drosophila* hematopoiesis. Curr Biol.

[15] Lundin V (2020). YAP regulates hematopoietic stem cell formation in response to the biomechanical forces of blood flow. Dev Cell.

[16] Jansson L, Larsson J (2012). Normal hematopoietic stem cell function in mice with enforced expression of the Hippo signaling effector YAP1. PLoS ONE.

[17] Donato E (2018). YAP and TAZ are dispensable for physiological and malignant haematopoiesis. Leukemia.

[18] Hao S (2019). Yap1 promotes proliferation of transiently amplifying stress erythroid progenitors during erythroid regeneration. Exp Hematol.

[19] Lorthongpanich C (2020). Effect of YAP/TAZ on megakaryocyte differentiation and platelet production. Biosci Rep.

[20] Lorthongpanich C (2017). The Hippo pathway regulates human megakaryocytic differentiation. Thromb Haemost.

[21] Roy A (2016). Uncoupling of the Hippo and Rho pathways allows megakaryocytes to escape the tetraploid checkpoint. Haematologica.

[22] Suraneni PK, Crispino JD (2016). The Hippo-p53 pathway in megakaryopoiesis. Haematologica.

[23] Damkham N (2022). YAP and TAZ play a crucial role in human erythrocyte maturation and enucleation. Stem Cell Res Ther.

[24] Griffiths RE (2012). Maturing reticulocytes internalize plasma membrane in glycophorin A-containing vesicles that fuse with autophagosomes before exocytosis. Blood.

[25] Laowtammathron C (2020). Derivation of human embryonic stem cell line MUSIe001-A from an embryo with homozygous alpha(0)-thalassemia (SEA deletion). Stem Cell Res.

[26] Thambyrajah R, Bigas A (2022). Notch signaling in HSC emergence: when, why and how. Cells.

[27] Butko E, Pouget C, Traver D (2016). Complex regulation of HSC emergence by the Notch signaling pathway. Dev Biol.

[28] Lampreia FP, Carmelo JG, Anjos-Afonso F (2017). Notch signaling in the regulation of hematopoietic stem cell. Curr Stem Cell Rep.

[29] Engel-Pizcueta C, Pujades C (2021). Interplay between notch and YAP/TAZ pathways in the regulation of cell fate during embryo development. Front Cell Dev Biol.

[30] Rayon T (2014). Notch and hippo converge on Cdx2 to specify the trophectoderm lineage in the mouse blastocyst. Dev Cell.

[31] Watanabe Y (2017). Notch and Hippo signaling converge on Strawberry Notch 1 (Sbno1) to synergistically activate Cdx2 during specification of the trophectoderm. Sci Rep.

[32] Xin Z (2021). Mapping human pluripotent stem cell-derived erythroid differentiation by single-cell transcriptome analysis. Genomics Proteomics Bioinformat.

[33] Hooglugt A (2020). Endothelial YAP/TAZ signaling in angiogenesis and tumor vasculature. Front Oncol.

[34] Nakajima H (2017). Flow-dependent endothelial YAP regulation contributes to vessel maintenance. Dev Cell.

[35] Sakabe M (2017). YAP/TAZ-CDC42 signaling regulates vascular tip cell migration. Proc Natl Acad Sci U S A.

[36] Uemura M, Nagasawa A, Terai K (2016). Yap/Taz transcriptional activity in endothelial cells promotes intramembranous ossification via the BMP pathway. Sci Rep.

