# Supplementary material for: Role of YAP in hematopoietic differentiation and erythroid lineage specification of human-induced pluripotent stem cells
Source: Stem Cell Res Ther. 2023 Sep 29;14:279. doi: 10.1186/s13287-023-03508-z (PMC10543272; doi:10.1186/s13287-023-03508-z)

# Supplementary Figure 1
# (Associated with figure 1B and C)

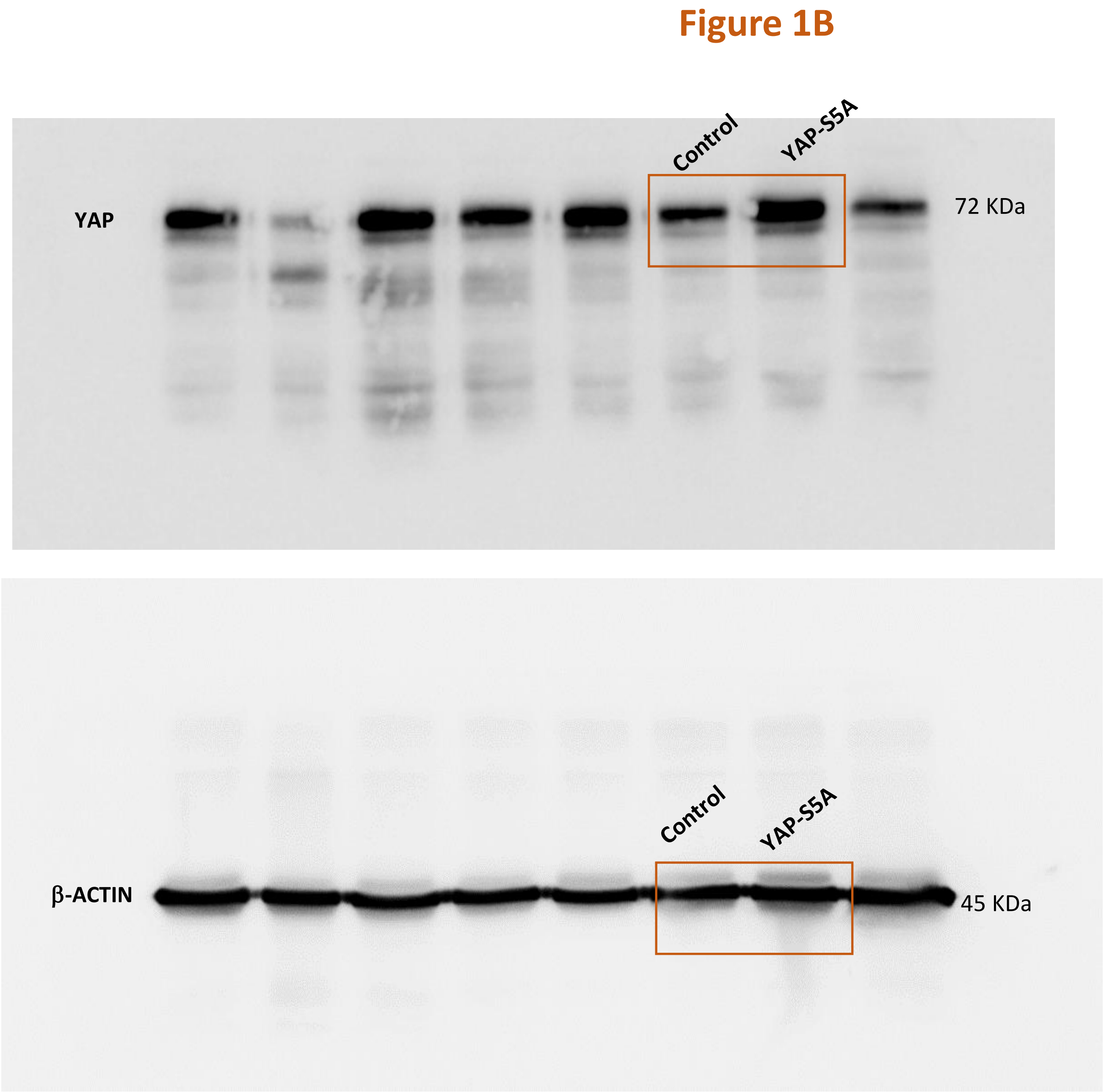

# Supplementary Figure 1
# (Associated with figure 1B and C)

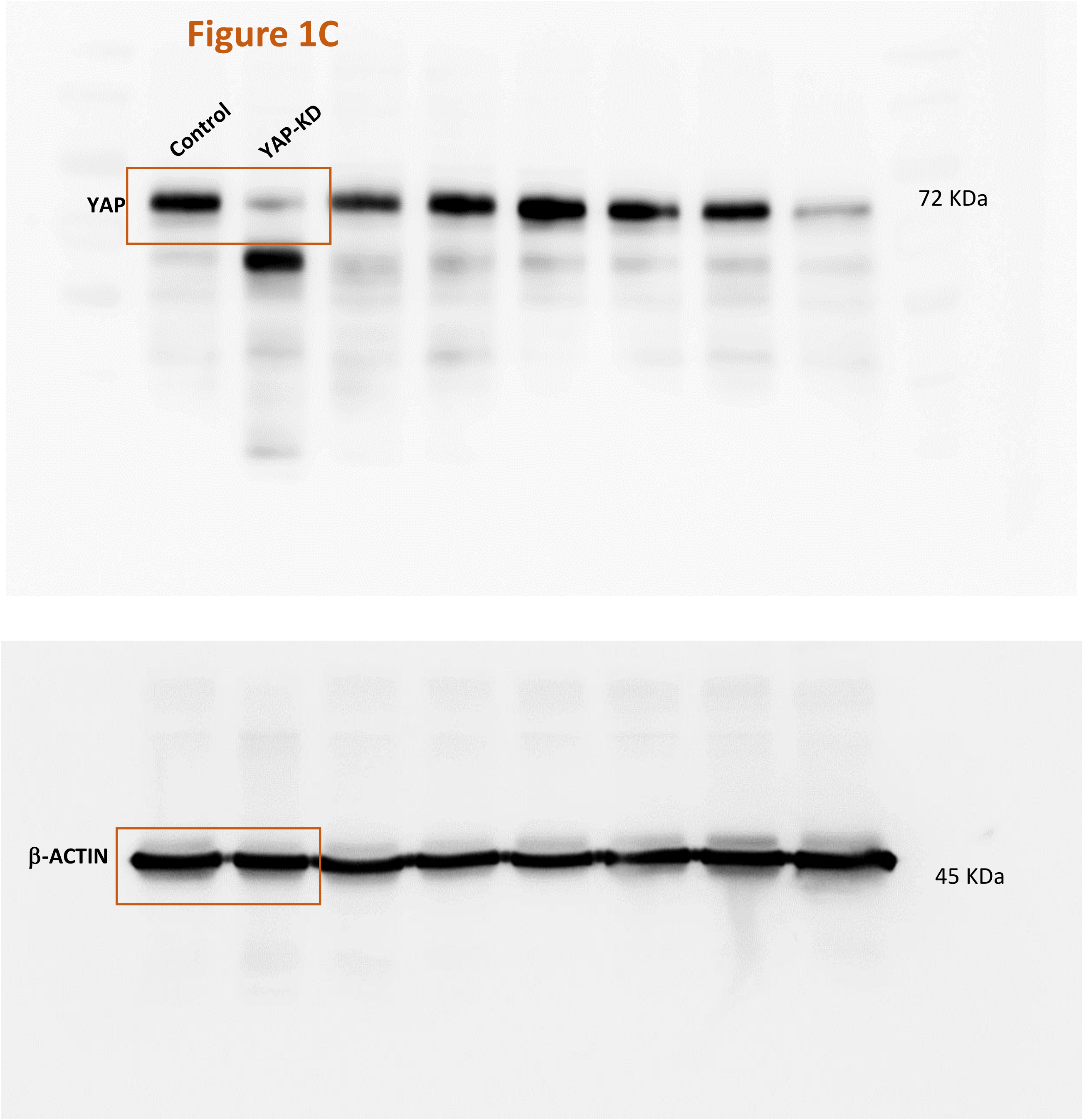

# Supplementary Figure 2A (Associated with figure 1F)

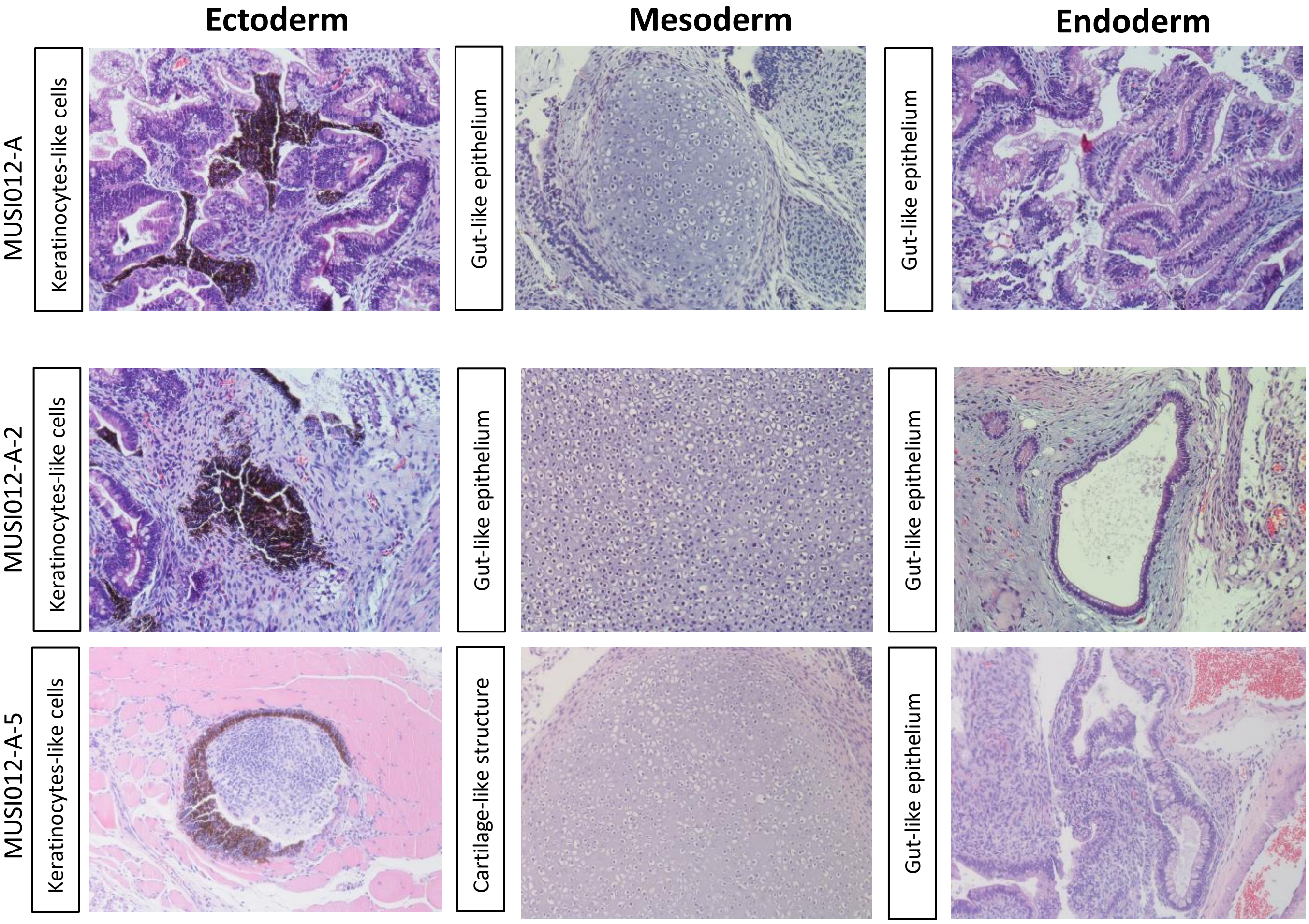

# Supplementary Figure 2B
# (Associated with figure 1F)

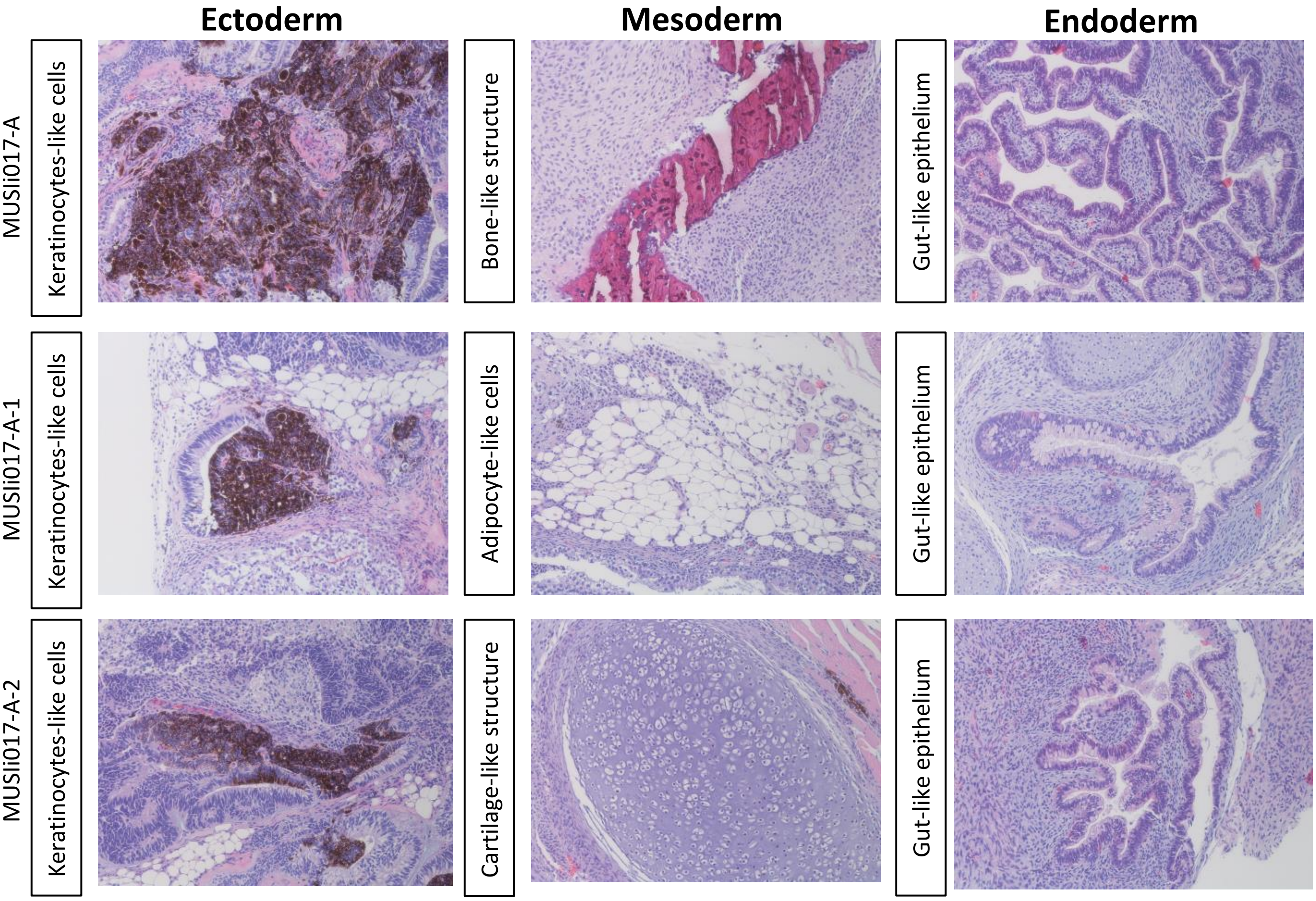

# Supplementary Figure 3
# (Associated with figure 4)

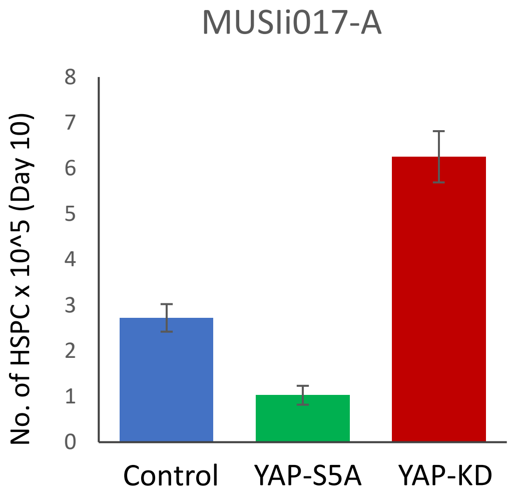

# Supplementary Figure 4

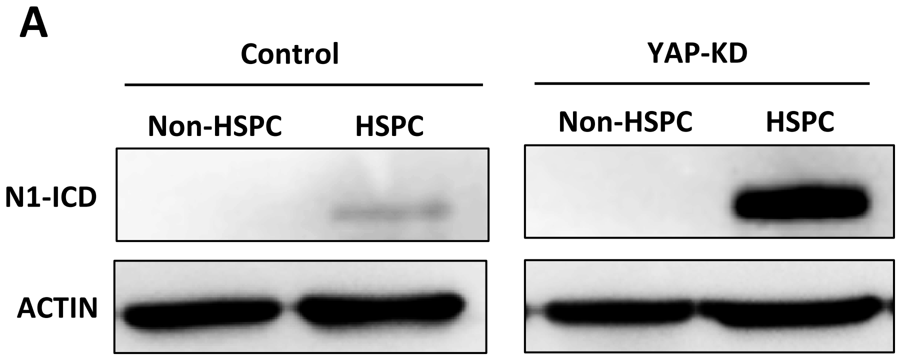


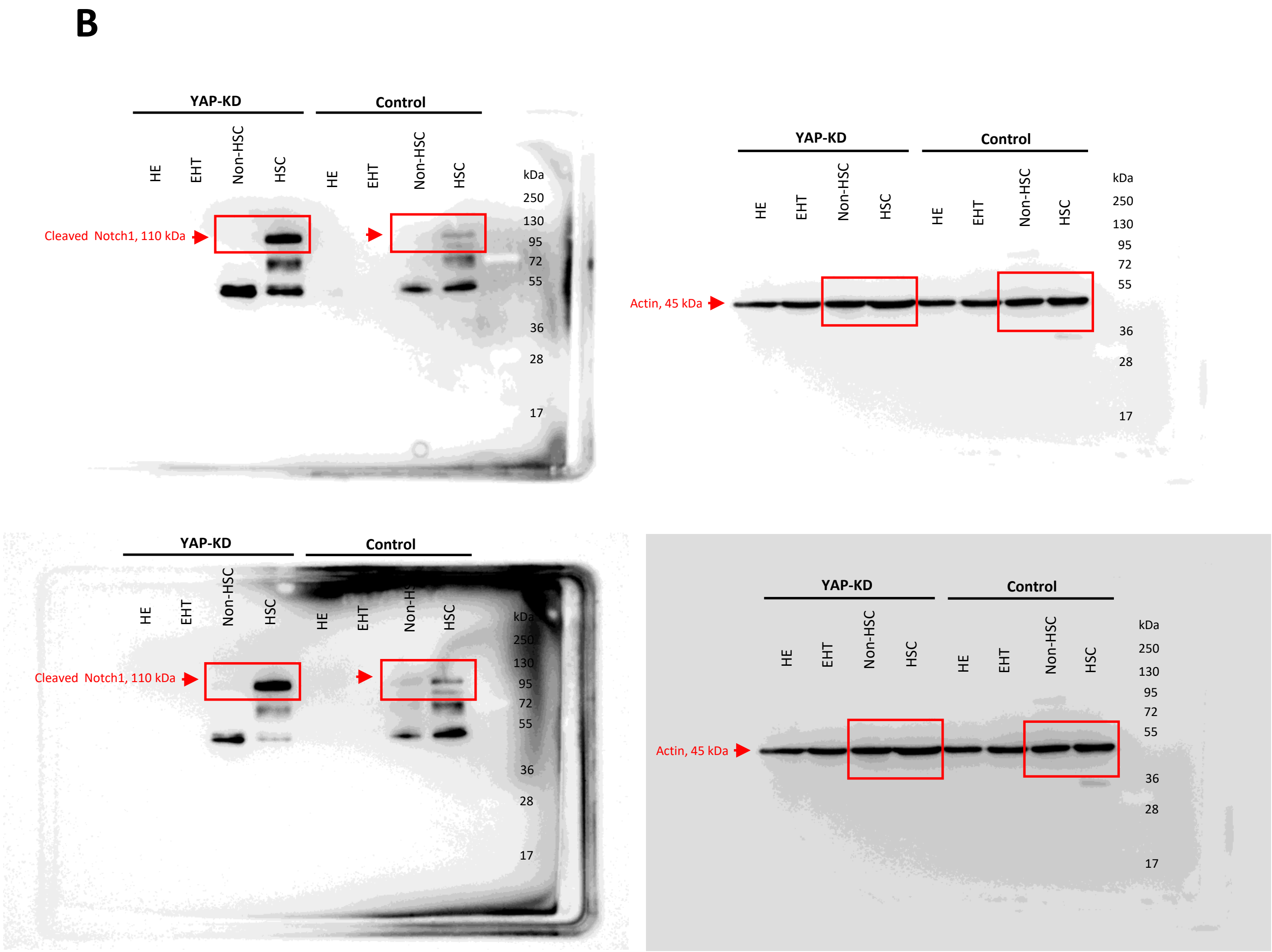

# Supplementary Figure 4

**C**

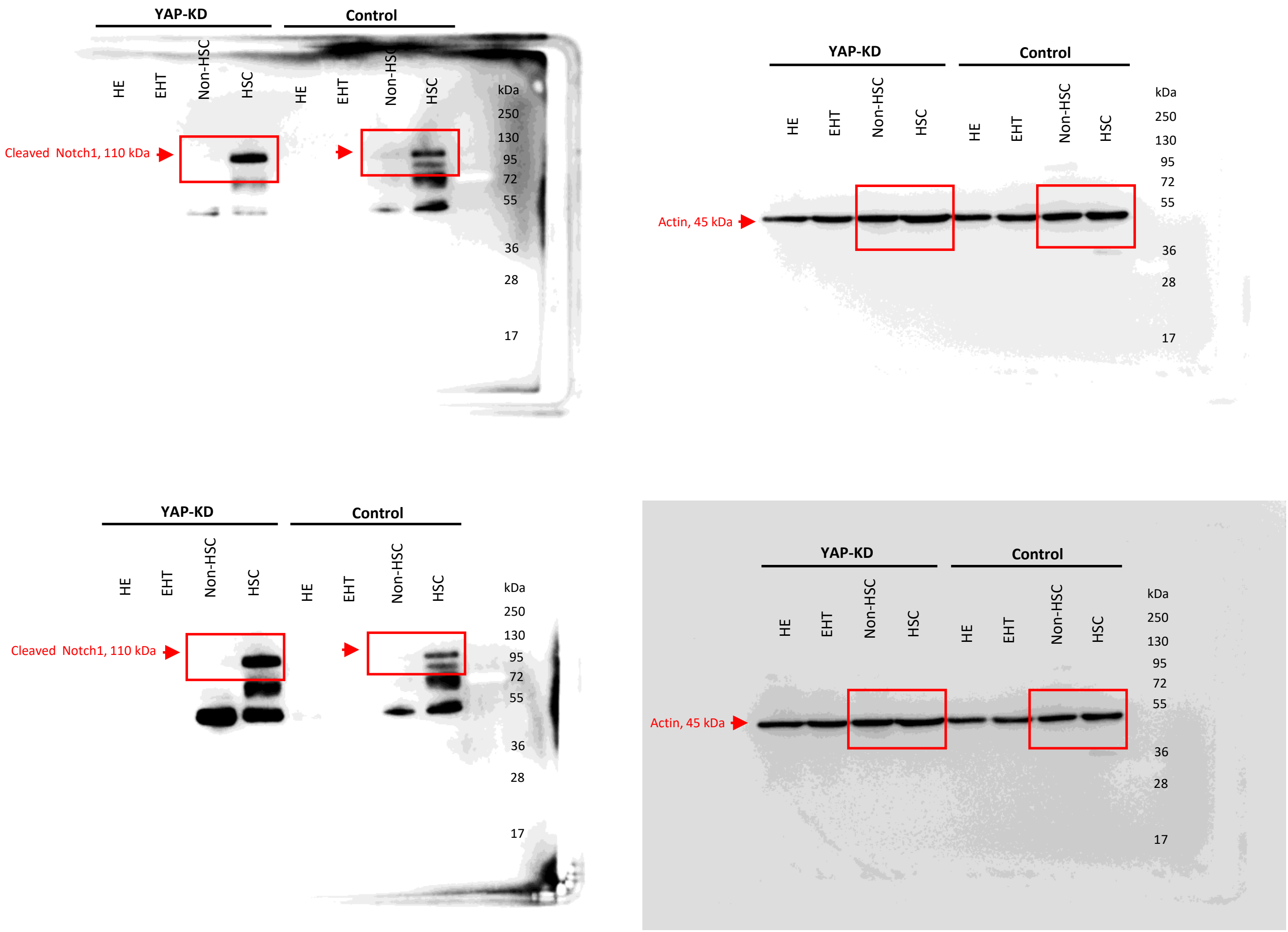

# Supplementary Figure 5 (Associated with figure 6G)

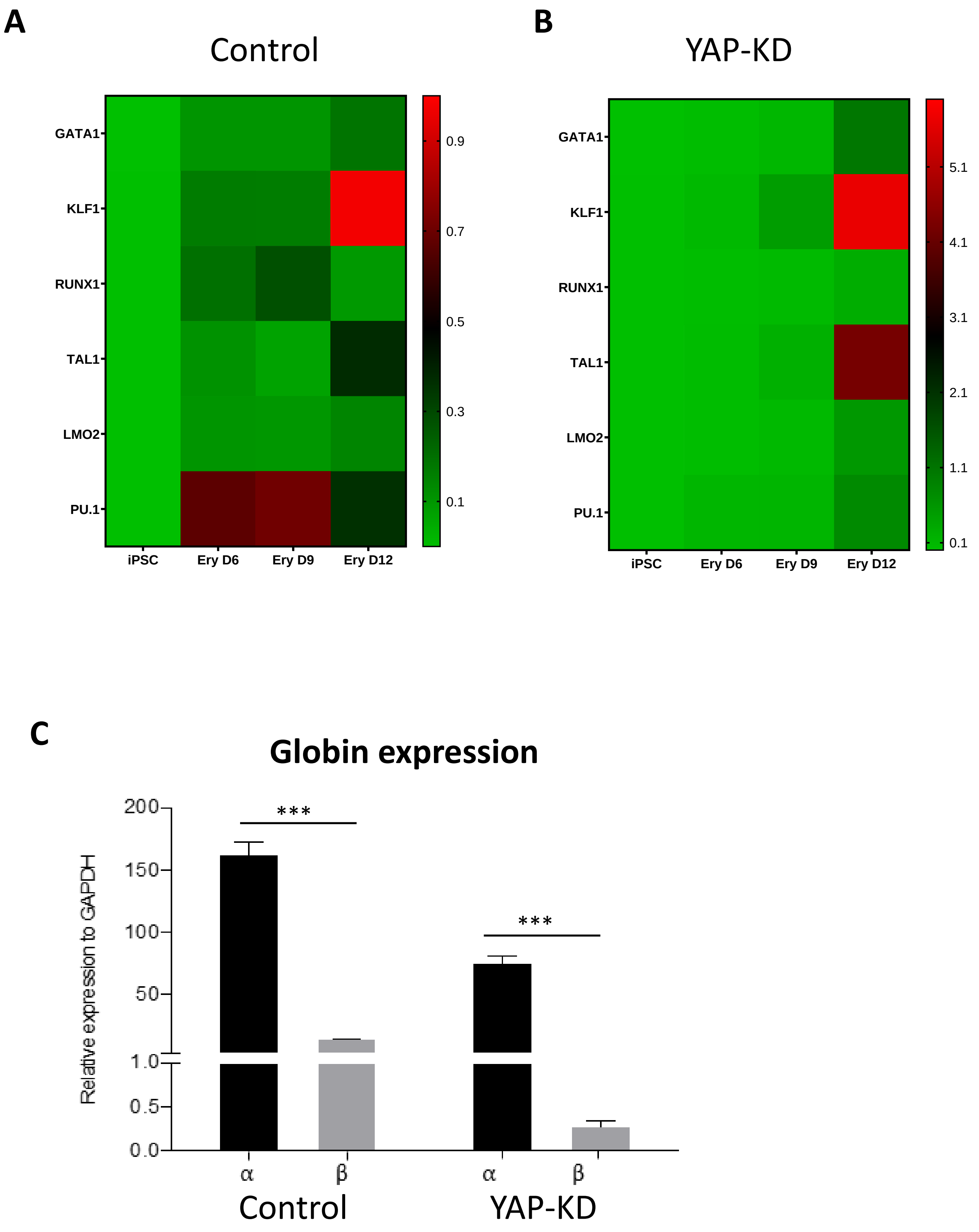

# Supplementary Figure 6
# (Associated with figure 6)

Megakaryocyte and platelets CD41+

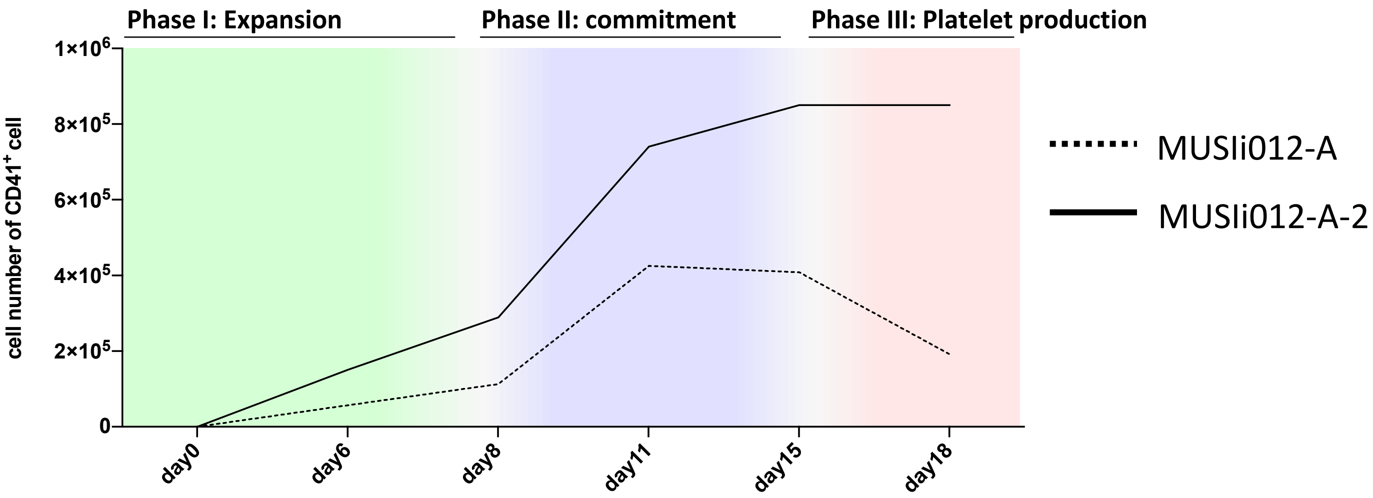

Supplement: Supplementary file 1 — Additional file 1: Fig. S1. A representative photograph of an entire western blot membrane probed with YAP and ACTIN specific antibodies. The areas indicate the results used in Figures 1B and 1C, respectively. Fig. S2. H&E stained images of teratoma derived from human iPSC lines used in this study. A and B) Staining of teratoma derived from MUSIi012-A and MUSIi017-A and their YAP-manipulated subclones to determine representative cells of the 3-embryonic germ layers, including ectoderm, mesoderm and endoderm. Fig. S3. Number of hematopoietic progenitor cell (HSPCs) produced from MUSIi017-A (control), MUSIi017-A-1 (YAP-KD), MUSIi017-A-2 (YAP-overexpressing) as determined at day 10 of differentiation. Fig. S4. A) Expression of N1-ICD in non-Hematopoietic Progenitor cell (Non-HSPCs) and HSPCs derived from MUSIi012-A control and MUSIi012-A-2 (YAP-KD) cell lines. B) Representative photographs of the entire western blot membrane probed with N1-ICD and ACTIN specific antibodies. (C) Multiple exposures of the same blot shown in A. The areas indicate the results used in supplementary figure 4A. Fig. S5. A and B) Heatmap showing the expression pattern of erythroid induction genes in control and YAP-KD HSPCs after cultured in erythroid differentiation media for 18 days. C) Relative expression of globin to GAPDH in erythroid derived-IPSCs, Data were collected from at least three times independently with the technical triplicate, and data were expressed as mean ± SEM, ***P < 0.001. Supplementary figure 6: Number of CD41+ cells megakaryocyte and platelets production from MUSIi012-A-2 (YAP-KD) iPSCs compared to MUSIi-012-A (control). The measurement was done at 6 timepoints during the differentiation in sequential differentiation medium. [file 13287_2023_3508_MOESM1_ESM.pdf]
