# Supplementary material for: Role of YAP in hematopoietic differentiation and erythroid lineage specification of human-induced pluripotent stem cells
Source: Stem Cell Res Ther. 2023 Sep 29;14:279. doi: 10.1186/s13287-023-03508-z (PMC10543272; doi:10.1186/s13287-023-03508-z)
Supplement: Supplementary file 2 — Additional file 2: Table S1. List of primers used in this study. [file 13287_2023_3508_MOESM2_ESM.pdf]

Supplementary Table 1. List of primer used in this study.

**Pluripotent gene**	**Forword**	**Reverse**
OCT-4	GAAGTTAGGTGGGCAGCTTG	TGTGGCCCCAAGGAATAGT
SOX2	GGGGGAATGGACCTTGTATAG	GCAAAGCTCCTACCGTACCA
NANOG	GAGATGCCTCACACGGAGAC	AGGGCTGTCCTGAATAAGCA
**Endoderm marker**		
ALB	GTGAGGTTGCTCATCGGTTT	GAGCAAAGGCAATCAACACC
FOXA2	CGTTCCGGGTCTGAACTG	ACCGCTCCCAGCATACTTT
Gata4	GGAAGCCCAAGAACCTGAAT	GTTGCTGGAGTTGCTGGAA
**Mesoderm marker**		
ACTC1	GCTTCCGCTGTCCTGAGA	ATGCCAGCAGATTCCATACC
MEF2g	TGATCAGCAGGCAAAGATTG	TGGACACTGGGATGGAGACT
T-brachury	TGCTTCCCTGAGACCCAGTT	GATCACTTCTTTCCTTTGCATCAAG
**Ectoderm marker**		
NEFH	CAGTCCGAGGAGTGGTTCC	GAGCGCATAGCGTCTGTGT
NESTIN	TGCGGGCTACTGAAAAGTTC	TGTAGGCCCTGTTTCTCCTG
PAX6	GGCACACACACATTAACACACTT	GGTGTGTGAGAGCAATTCTCAG
**Hematopoietic lineage**		
MYB	GCCACTTCCCTAACCGCAC	CCCTTGACAAGGTCTGGATTC A
RUNX1C	GAAGTCTGAACCCAGCATAGTGGTCAGC	GTGGACGTCTCTAGAAGGATTCATTCCAAG
TAL1	GTACCCCCGTAGCGGAAA	AGGTGTCTACGCGGTTGC
PU1	CACAGCGAGTTCGAGAGCTT	GATGGGTACTGGAGGCACAT
MEIS1	GCCATACAAGTGTTAAGGTTTCATC	CCTCCTTCTCTATCATCTATCACAAA
**Endothelium lineage**		
ETV2	CCGACGGCGATACCTACTG	CGGTGGTTAGTTTTGGGGCAT
FLK1	TTCTGGACTCTCTCTGCCTACC	AGAACCATACCACTGTCCGTCT
SOX17	GTGGACCGCACGGAATTTG	GAGGCCCATCTCAGGCTTG
**Erythroid marker**		
GATA1	CAC TGA GCT TGC CAC ATC C	ATG GAG CCT CTG GGG ATT A
GATA2	TTG TGC AAA TTG TCA GAC GAC	TCA TGG TCA GTG GCC TGT TA
KLF1	TTA CGG AAA ATC CGA CAA GC	TGC ACG ACA GTT TGG ACA TC
LMO2	CGA GGA CTG CCT GAG CTG	AGA CCG TCT TGC CCA AAA A
BCL11A	CCA AAC AGG AAC ACA TAG CAG A	GAG CTC CAT GTG CAG AAC G
