# Supplementary material for: Role of YAP in hematopoietic differentiation and erythroid lineage specification of human-induced pluripotent stem cells
Source: Stem Cell Res Ther. 2023 Sep 29;14:279. doi: 10.1186/s13287-023-03508-z (PMC10543272; doi:10.1186/s13287-023-03508-z)
Supplement: Supplementary file 3 — Additional file 3: Table S2. List of antibodies used in this study. [file 13287_2023_3508_MOESM3_ESM.pdf]

Supplementary Table 2 List of antibodies used in this experiment.

Antibody	Catalog no.	Company
**Antibodies for HSC isolation**		
anti-human CD34-PE	#343506	BioLegend, San Diego, CA, USA
anti-human CD43-PE/cyanine7	#343208	BioLegend, San Diego, CA, USA
anti-human CD45-PerCP	#347464	Bioscience
**Antibodies used for early erythroid identification**		
anti-human CD235a-FITC	#349104	BioLegend, San Diego, CA, USA
anti-human CD41-APC	#349116	BioLegend, San Diego, CA, USA
**Antibodies used for T/B cell differentiation**		
anti-human TRA-1-60-PE	#330609	BioLegend, San Diego, CA, USA
anti-human CD10-PerCP/cyanine5.5	#312216	BioLegend, San Diego, CA, USA
anti-human CD3-FITC	#300306	BioLegend, San Diego, CA, USA
anti-human CD19-FITC	#302206	BioLegend, San Diego, CA, USA
**Antibody used for granulocyte differentiation**		
anti-human CD33-PE/cyanine7	#366618	BioLegend, San Diego, CA, USA
